# Transcriptional regulation of INK4/ARF locus by cis and trans mechanisms

**DOI:** 10.3389/fcell.2022.948351

**Published:** 2022-09-09

**Authors:** Umer Farooq, Dimple Notani

**Affiliations:** ^1^ Genetics and Development, National Centre for Biological Sciences, Tata Institute for Fundamental Research, Bangalore, India; ^2^ The University of Trans-Disciplinary Health Sciences and Technology, Bangalore, India

**Keywords:** INK4/ARF, enhancer, 9p21, gene desert, p15^INK4b^, p16^INK4a^, ANRIL, CDKN2BAS

## Abstract

9p21 locus is one of the most reproducible regions in genome-wide association studies (GWAS). The region harbors *CDKN2A/B* genes that code for p16^INK4a^, p15^INK4b^, and p14^ARF^ proteins, and it also harbors a long gene desert adjacent to these genes. The polymorphisms that are associated with several diseases and cancers are present in these genes and the gene desert region. These proteins are critical cell cycle regulators whose transcriptional dysregulation is strongly linked with cellular regeneration, stemness, aging, and cancers. Given the importance of this locus, intense scientific efforts on understanding the regulation of these genes via promoter-driven mechanisms and recently, via the distal regulatory mechanism have provided major insights. In this review, we describe these mechanisms and propose the ways by which this locus can be targeted in pathologies and aging.

## Introduction

**FIGURE 1:**
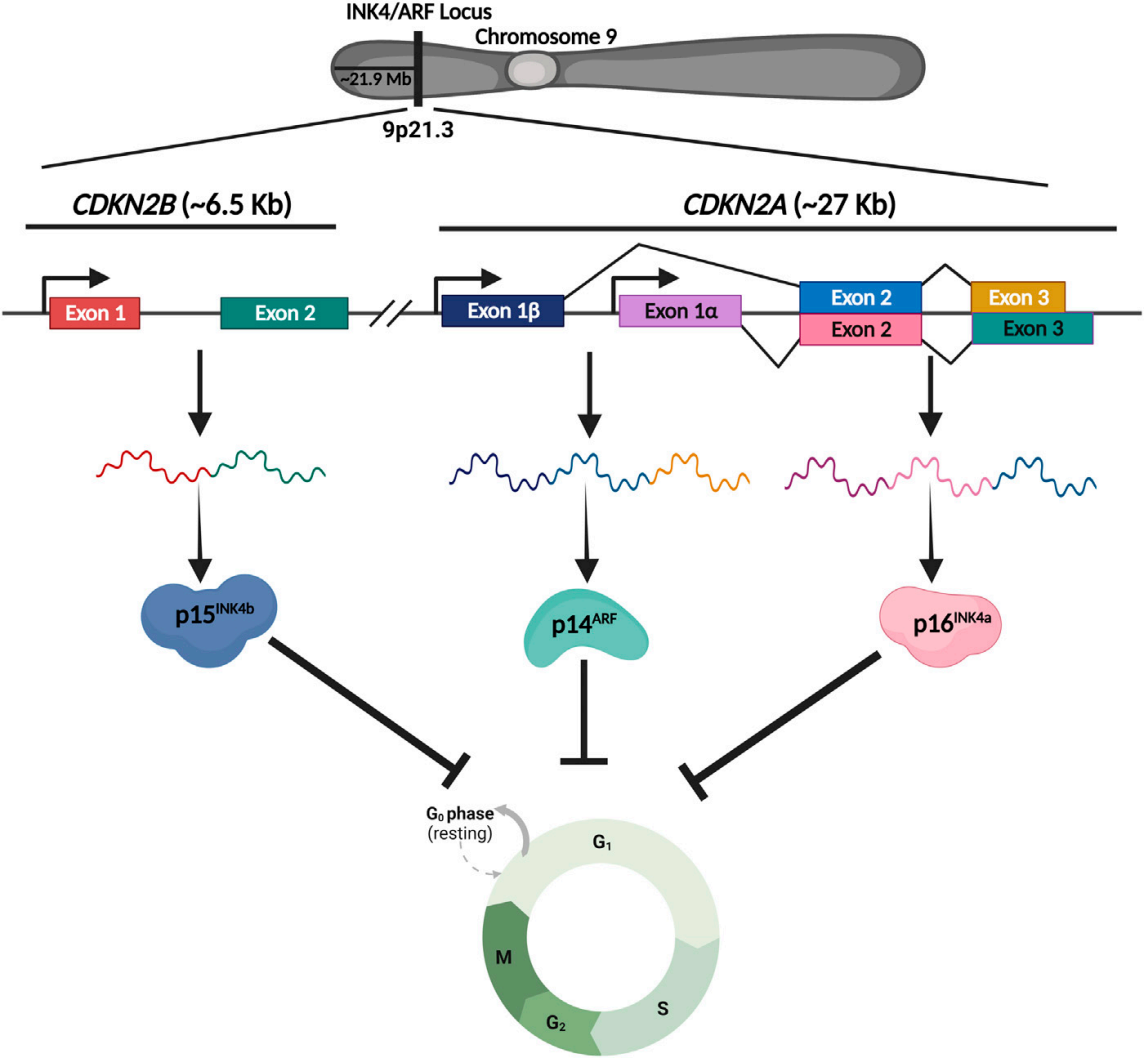
Schematic representing the genomic structure of INK4/ARF locus. INK4/ARF locus harbors two genes, *CDKN2A* and *CDKN2B*, that code for three critical cell cycle regulators. *CDKN2A* gene produces two proteins, p16^1NK4a^ and p14^ARF^, whereas the *CDKN2B* gene produces p15^1NK4b^. Together these genes regulate the cell cycle under various conditions.

The INK4/ARF locus functions are attributed to three distinct but related proteins, namely, p14^ARF^, p16^INK4a^, and p15^INK4b^. These proteins are coded by two genes; *CDKN2A* and *CDKN2B*. p14^ARF^ and p16^INK4a^ are transcribed from the *CDKN2A* gene, whereas p15^INK4b^ is transcribed from the *CDKN2B* gene (Figure 1). The initial exons of p14^ARF^ (exon1β) and p16^INK4a^ (exon1α) are different, but the second and third exons are identical. While the mRNA sequences of p14^ARF^ and p16^INK4a^ are relatively similar, the resultant proteins do not share any sequence similarity due to the alternative reading frames; thus, these proteins are not isoforms. On the other hand, p15^INK4b^ and p16^INK4a^ have a high degree of amino acid similarity (about 80%) and are thought to have emerged from a gene duplication event (Lopez et al., 2017). Additionally, there is a *CDKN2BAS* gene that transcribes a non-coding RNA known as ANRIL. Because ANRIL is transcribed in the antisense direction relative to *CDKN2B*, the gene is termed *CDKN2BAS*. Together, these proteins regulate the cell cycle progression and are known to operate as a barrier to the reprogramming of somatic cells. Inactivation of this locus due to homozygous deletions or epigenetic alterations such as transcriptional silencing by DNA methylation or polycomb-mediated suppression is a frequent event that occurs in a wide spectrum of cancers. Furthermore, single nucleotide polymorphisms (SNPs) in this locus are associated with several aging-related disorders, including coronary artery disease (CAD), type 2 diabetes, and atherosclerosis. The majority of the SNPs in this locus are located within the genes and the ∼0.3 Mb long adjacent gene desert region, but the mechanisms of their action are largely unknown. Thus, the identification of molecular pathways that regulate this locus in different diseases is of great therapeutic relevance.

## Cell cycle regulation by INK4/ARF proteins

**FIGURE 2:**
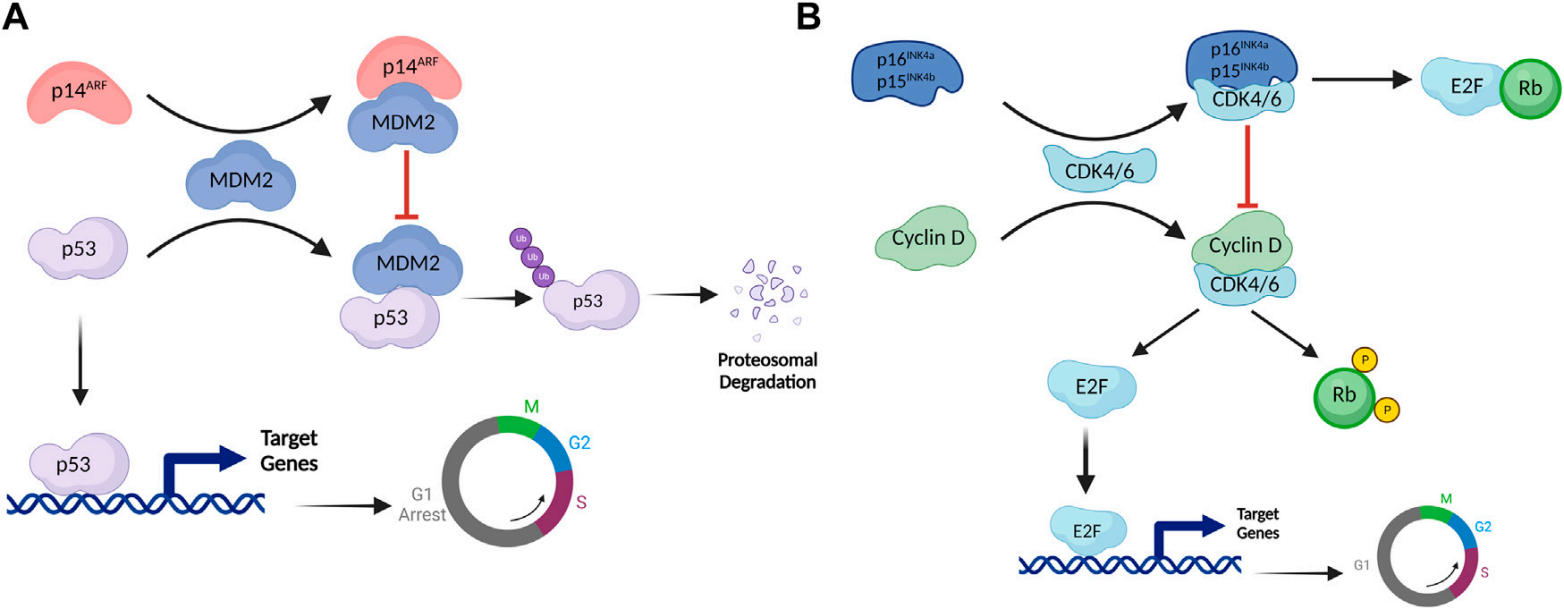
The INK4/ARF cell cycle regulatory network. **(A)** The p53-MDM2 pathway is controlled by the upstream effector protein p14^ARF^. By establishing a complex with MDM2, p14^ARF^ permits p53 to activate its transcriptional targets. MDM2 ubiquitinates p53, which mediates proteasomal degradation in normal conditions. However, when MDM2 interacts with p14^ARF^, NoLS of MDM2 is exposed, resulting in MDM2 sequestration in the nucleolus. MDM2 sequestration prevents degradation of p53, allowing it to activate its transcriptional targets and arrest the cell cycle in the G1 phase. **(B)** The retinoblastoma pathway is regulated by p16^INK4a^ and p15^INK4b^. E2F is a transcription factor that activates genes involved in the transition from G1 to M phase. Rb inhibits this function of E2F by establishing a complex with it. Under normal conditions, the cyclin D-CDK4/6 complex phosphorylates Rb. Phosphorylated Rb doesn't engage with E2F, as a result, E2F binds to target genes to activate them. Once expressed, p16^INK4a^/p15^INK4b^ inhibits cyclin D-CDK4/6 complex formation, keeping Rb hypophosphorylated. Hypophosphorylated Rb forms a complex with E2F, inhibiting its transcriptional activity.

Several stress signals including oncogene overexpression, DNA damage, oxidative stress, etc., induce the expression of *INK4/ARF* genes (Romagosa et al., 2011). Once activated, these genes trigger a cascade of signaling events that effectively bring the cell cycle to a halt (Ivanchuk et al., 2001). Mechanistically, p53 (a well-studied tumor suppressor that blocks the cell cycle at the G1 phase) is a downstream effector of the p14^ARF^ pathway (Sherr 2001). The interaction of MDM2 with p53 alters the stability and cellular localization of p53 (Kubbutat, Jones, and Vousden 1997). MDM2 acts as an E3 ubiquitin ligase and mediates the proteasomal degradation of p53 by ubiquitinating its C-terminal domain (Wade, Wang, and Wahl 2010). Multiple domains of p53 interact with MDM2, including the DNA binding domain (DBD), the transactivation domain (TAD), and the carboxy-terminal domain (CTD). MDM2, on the other hand, interacts with p53 via its N-terminal hydrophobic domain (HD) and acid domain (AD) (Chi et al., 2005; Yu et al., 2006; Poyurovsky et al., 2010). When expressed, p14^ARF^ interacts with the acid domain of MDM2, preventing it from interacting with p53. This interaction alters the conformation of MDM2 that exposes its Nucleolar localization signal (NoLS) present in the RING domain (RD), leading to sequestration of the MDM2-p14^ARF^ complex in the nucleolus (Weber et al., 1999; Maggi et al., 2014). Sequestration of MDM2 in the nucleolus prevents MDM2-mediated export of p53 to the cytoplasm, hence preventing its degradation (Maggi et al., 2014). These events lead to p53 translocation into the nucleus thereby, activating genes that cause the cell cycle to arrest at the G1 phase (Weber et al., 1999) (Figure 2A).

p16^INK4a^ and p15^INK4b^, on the other hand, regulate the retinoblastoma (Rb) pathway. These proteins activate Rb, a tumor suppressor protein that blocks the cell cycle at the G1 phase (Kim and Sharpless 2006). CDK4/6 typically forms an active complex with cyclin D that binds to and phosphorylates Rb (Cobrinik 2005). Rb loses its ability to interact with the E2F transcription factor in the phosphorylated state (Dimova and Dyson 2005). E2F activates genes involved in the cell cycle transition from G1 to S (Giacinti and Giordano 2006). When stress signals activate p16^INK4a^/p15^INK4b^, these proteins bind to CDK4/CDK6, causing an allosteric shift in the latter proteins, preventing them from forming the active complex with cyclin D, thereby maintaining Rb in a hypophosphorylated state (Hannon and Beach 1994; Russo et al., 1998). Hypophosphorylated Rb binds with the transactivation domain of E2; this complex subsequently recruits HDAC1 and SUV39H1 to the E2F target genes, thereby inhibiting them and preventing the G1 to S phase transition (Giacinti and Giordano 2006) (Figure 2B). These proteins being high in cellular senescence, permanently inhibit cell division. However, HPV-positive cancer cells express significant levels of p16^INK4a^, p14^ARF^, and p15^INK4b^ without undergoing cell cycle arrest, attributed to two HPV-encoded oncoproteins, E6 and E7 (Kanao et al., 2004). These proteins inhibit the downstream effectors of p14^ARF^ and p16^INK4a^ genes, thereby preventing cell cycle arrest. E7 interacts with Rb, leading to its inactivation, whereas E6 induces the degradation of p53 protein (Munger et al., 1992).

## Implication of INK4/ARF locus in aging, cancer, and regeneration

### INK4/ARF locus in senescence/aging

Senescence is an innate cellular response in which normally proliferating cells cease to divide permanently in response to specific intrinsic and extrinsic stimuli. Senescent cells exhibit morphological and physiological changes, the formation of senescence-associated heterochromatin foci (SAHF), and the release of senescence-associated secretory phenotype (SASP), etc., (van Deursen 2014). This irreversible cell cycle halt is thought to be the first line of defense against cancer by preventing the division of abnormal cells (Prieto and Baker 2019). Senescence, on the other hand, plays a significant role in aging-related pathologies, as it impairs tissue repair and regeneration (McHugh and Gil 2018). Several recent studies have expanded our understanding of the role of senescence in other complex biological processes such as development, and tissue repair, among others (Herranz and Gil 2018). p16^INK4a^ is the fundamental driver and a well-established biomarker of senescence (Krishnamurthy et al., 2004; Rayess, Wang, and Srivatsan 2012). Studies have demonstrated that ectopic expression of oncogenes like Ras and Raf, increases p16^INK4a^ expression, triggering premature senescence in various cell types (Lin et al., 1998; Zhu et al., 1998). For example, fibroblasts, epithelial cells, and T lymphocytes, express higher p16^INK4a^ when they approach replicative senescence (Lin et al., 1998; Zhu et al., 1998; Mirzayans et al., 2012). In summary, the INK4/ARF locus regulates oncogene-induced and replicative senescence in several cell types (Mirzayans et al., 2012).

### INK4/ARF locus in cancer

Cancer cells proliferate abnormally and do not respond to signals that regulate cell growth and division. Most frequently, cancer cells contain mutations in genes that regulate the cell cycle; once altered, these genes lose their ability to control the cell cycle (Papp and Plath 2011). As mentioned previously, *INK4/ARF* genes are cell cycle regulators that arrest the cell cycle at various stages in response to stress signals such as DNA damage. These tumor suppressor genes must be silenced for cancer to progress. Thus, INK4/ARF locus harbors homozygous deletions in several malignancies, silencing the expression of all three cell cycle regulator genes (Sherr 2012). Similarly, loss of p16^INK4a^ expression through specific point mutations has been reported in several cancers (Forbes et al., 2006). The suppression of this locus by DNA hypermethylation at the promoters or through histone modifications mediated by the PRC2 complex is also prevalent in cancers. In animal studies, mice lacking either *INK4a* or *ARF* gene are more susceptible to certain tumors than mice lacking the *INK4b* gene. On the other hand, overexpression of the *INK4/ARF* genes results in a threefold reduction in tumor incidence in mice (Matheu et al., 2004).

### INK4/ARF locus in cellular reprogramming

Cellular plasticity facilitates the reprogramming of somatic cells to a more pluripotent state. This reprogramming process considerably alters the epigenetic and chromatin landscapes of the cells (Papp and Plath 2011). A few critical transcription factors, like Oct4, Sox2, Klf4, Nanog, and others, can transform a somatic cell into a pluripotent cell (Papp and Plath 2011). However, the primary limitation of reprogramming is its significantly lower efficiency (approx. 1%). In the fast-dividing embryonic stem cells and induced pluripotent stem cells (iPSCs), the INK4/ARF locus is repressed. This locus, however, is activated during the reprogramming process as a result of highly mitogenic cell culture conditions (Sharpless 2005). As a result of the activation of this locus in somatic cells, reprogramming efficiency decreases significantly. Conversely, mouse embryonic fibroblasts (MEFs) lacking the INK4/ARF locus reprogram more efficiently with 15-fold higher efficiency (Li et al., 2009). While silencing *INK4a* or *ARF* alone improves reprogramming efficiency, double silencing results in increased efficiency, as seen in *INK4/ARF* null cell lines (Li et al., 2009). Not only is the efficiency increased, but the rate at which iPSC colonies develop is also increased in *INK4/ARF* defective cells. Interestingly, *ARF* is the primary regulator of cell reprogramming in murine cells, but *INK4a* is the dominant regulator in humans (Li et al., 2009).

## Transcriptional regulation of INK4/ARF locus

### Repression of INK4/ARF locus *via* PRC complexes

Polycomb group (PcG) proteins are epigenetic modifiers that play a crucial role in transcriptional repression and therefore regulate cell proliferation, differentiation, embryonic development, cellular memory, and other vital cellular functions (Wang et al., 2015). PcGs form two major protein complexes, the Polycomb repressive complex 1 (PRC1) and the Polycomb repressive complex 2 (PRC2). PRC2 exerts inhibition by adding trimethyl marks to lysine 27 of histone 3 (H3K27me3). The trimethyl mark serves as a docking site for PRC1, which recognizes this mark and monoubiquitinates Histone 2A at lysine 119 (H2AK119ub) (Chittock et al., 2017). The H2AK119ub further enhances H3K27me3 deposition by PRC2 and subsequent recruitment of PRC1 (Chittock et al., 2017). Both PRC1 and PRC2 are multimeric protein complexes with several core subunits and a few auxiliary subunits (Kerppola 2009). The PRC1 core consists of RING1A/B, PCGF2/4, CBX2/4/6/7/8, PHC1/2/3 subunits, while PRC2 contains Suz12, Ezh2, RbAp46/48, and Eed as core subunits (Chittock et al., 2017). Additionally, various auxiliary subunits aid or improve the activity of these complexes (Chittock et al., 2017). Ezh2, a SET domain-containing protein, is the enzymatic component of the PRC2 complex responsible for the trimethylation of H3K27. At the INK4/ARF locus, PcG inhibits the promoters by trimethylating H3K27 to increase cell proliferation (Bracken et al., 2007). PcGs have been demonstrated to repress all three *INK4a*, *ARF*, and *INK4b* genes in some instances, but only *INK4a* and *INK4b* in others (Bruggeman et al., 2005; Bracken et al., 2007; Kheradmand Kia et al., 2009). Ectopic expression of PcG subunits such as Bmi1, Ezh2, CBX7, and CBX8 has been shown to downregulate *INK4a* and *INK4b* expression to bypass senescence (Jacobs et al., 1999; Gil et al., 2004; Dietrich et al., 2007). In contrast, depletion of the PcG subunits activates this locus, resulting in cell growth inhibition and senescence (Bracken et al., 2007; Dietrich et al., 2007).

Several transcription factors facilitate PcG binding to *INK4/ARF* promoters; for example, Zfp277, a zinc finger protein, interacts with the Bm1 subunit of PRC1 to recruit PRC1 to these promoters in MEFs (Negishi et al., 2010). Zfp277 depletion causes the PRC1 complex to displace from the promoters, activating *INK4a/ARF* gene and early senescence (Negishi et al., 2010). Similarly, Homeobox proteins such as HLX1 and HOXA9 play an essential role in suppressing *INK4a*. These proteins cooperate with PRC2 and HDACs at the *INK4a* promoter to mediate the repression (Martin et al., 2013). Haematopoietically expressed homeobox gene (Hhex) is vital in maintaining acute myeloid leukemia (AML), as its deletion causes upregulation of *INK4a* and *ARF*. Further, Hhex, like HLX1 and HOXA9, facilitates PRC2 binding to the promoters by interacting with the Suz12 subunit, thereby repressing the genes (Shields et al., 2016). In neonatal human diploid fibroblasts (HDFs), PRC2 binding to the *INK4a* promoter and the upstream region of the *INK4b* promoter induces a long-range interaction (repressive chromatin loop) between these promoters (Kheradmand Kia et al., 2009). Similar long-range interaction between the *INK4a* and *INK4b* promoters has been observed in hematopoietic progenitor cells and malignant rhabdoid tumors (MRTs) (Kheradmand Kia et al., 2009). In mature HDFs, however, the chromatin architecture of these genes is noticeably different where the looping between *INK4a* and *INK4b* is lost. Under such alterations, transcriptional activation and senescence induction occurs due to the concomitant loss of Ezh2 binding on promoters (Kheradmand Kia et al., 2009).

### JMJD3-mediated transcriptional activation of INK4/ARF locus

Jumonji domain-containing D3 protein (JMJD3) is a lysine-specific histone demethylase. Its role in development, cancer progression, infectious diseases, immune disorders, and other conditions has been extensively studied (Xiang et al., 2007; Zhang X et al., 2019). JMJD3 belongs to the Jumonji (JmjC) domain-containing protein family, and this domain enzymatically catalyzes the removal of trimethyl marks from Histone 3 at lysine 27 (H3K27me3). Ubiquitously transcribed TPR protein on the X chromosome (UTX) is another demethylase that also demethylates H3K27me3 (Agger et al., 2007). While UTX is ubiquitously expressed, JMJD3 is induced in response to certain signaling events such as stress, etc (Swigut and Wysocka 2007). Due to its antagonistic role relative to PcG proteins, JMJD3 is a positive regulator of the *INK4/ARF* during the onset of cellular senescence (Agger et al., 2009; Barradas et al., 2009). Many cellular signals have been implicated in the induction of JMJD3 expression and subsequent activation of the *INK4/ARF* genes. For example, oncogene-mediated upregulation of JMJD3 causes activation of *INK4/ARF* genes in various cell types like fetal lung fibroblasts (IMR90), MEFs, etc., which results in INK4a-mediated growth arrest in these cells (Agger et al., 2009). By activating the INK4/ARF locus, JMJD3 prevents Schwann cells from proliferating uncontrollably in response to tumorigenic signals or following injury (Gomez-Sanchez et al., 2013). Under these conditions, JMJD3 binds to and demethylates the *INK4/ARF* promoters, activating these genes and initiating senescence. These cells lose the cell cycle control and continue to proliferate, resulting in neurofibromas when this pathway is disturbed (Gomez-Sanchez et al., 2013). As mentioned in previous section, INK4/ARF locus functions as a barrier to MEFs and keratinocyte reprogramming, and its silencing enhances reprogramming efficiency (Li et al., 2009). JMJD3 increases p16^INK4a^ and p14^ARF^ expression, limiting MEF reprogramming (Zhao et al., 2013). Therefore, JMJD3 silencing inhibits INK4/ARF-mediated cellular senescence, improving reprogramming efficiency. Moreover, double knockdown of JMJD3 and INK4a or ARF further enhances the reprogramming efficiency (Zhao et al., 2013).

### KDM2B-mediated transcriptional repression of INK4/ARF locus

KDM2B is an epigenetic modifier that preferentially demethylates trimethylated lysine 4 (H3K4me3) and dimethylated lysine 36 of histone H3 (H3K36me2) (Frescas et al., 2007). It regulates numerous biological processes, including cellular senescence, differentiation, and stem cell self-renewal (He et al., 2008; Liang et al., 2012; He et al., 2013). Furthermore, it is highly expressed in various cancers and plays a crucial role in cancer progression, especially in leukemia (Yan et al., 2018). KDM2B associates with the promoters of the *INK4/ARF* genes and demethylates histones H3K36me2 and H3K4me3. Demethylation results in a decrease in PolII binding and an increase in H3K27me3. KDM2B suppresses this locus by epigenetic modifications of histones and also by preventing the downregulation of Ezh2 (Tzatsos et al., 2009). Consequently, KDM2B protects MEFs from replicative and oncogenic senescence, and its knockdown decreases proliferation and induces senescence. Another study showed that KDM2B functions as a proto-oncogene and inhibits senescence by negatively regulating *INK4b*. Similarly, KDM2B achieves repression of *INK4b* by removing the active H3K36me2 mark near the promoter and the gene body, whereas its knockdown causes increased expression of *INK4b* (He et al., 2008).

### DNA methylation-mediated transcriptional repression of INK4/ARF locus

In addition to the aforementioned mechanisms, DNA methylation is another epigenetic mechanism to silence *INK4/ARF* genes. DNA methylation is catalyzed by DNA methyltransferases (Dnmts), which transfer the methyl group from S-adenosyl methionine (SAM) to carbon 5 of cytosine to generate 5-methylcytosine (5mC) (Lyko 2018). CpG islands in the promoters of tumor suppressor genes undergo abnormal hypermethylation in cancers (Robertson and Jones 1998). Notably, *INK4a* was one of the first genes discovered to be silenced in human cancers as a result of DNA methylation (Esteller et al., 2001). Numerous malignancies have been linked to aberrant CpG island methylation in the promoter region of the *INK4/ARF* genes. CpG islands are present near the promoter of *ARF* and exon1α of *INK4a* (Robertson and Jones 1998). Aberrant methylation of the *ARF* promoter is more prevalent than *INK4a* (Dominguez et al., 2003). Numerous types of cancer, including colon cancer, Merkel cell carcinoma, breast cancer, bladder tumors, and oligodendrogliomas, harbor abnormal DNA methylation of these genes (Watanabe et al., 2001; Tsujimoto et al., 2002; Lee et al., 2006; Lassacher et al., 2008).

### Chromatin remodelling of INK4/ARF locus *via* SWI/SNF complex

SWI/SNF is a multi-subunit ATP-dependent complex. This complex is largely involved in chromatin remodelling, which facilitates gene transcription by allowing transcription factors to access their DNA binding sites (Wilson and Roberts 2011). Abnormal expression and mutations in the SWI/SNF components can cause cancer (Klochendler-Yeivin, Muchardt, and Yaniv 2002; Orlando et al., 2019). Malignant rhabdoid tumors (MRTs) exhibit the loss of the hSNF5 gene, which encodes one of the subunits of the SWI/SNF complex (Biegel et al., 1999; Sevenet et al., 1999; Roberts and Orkin 2004). On the other hand, ectopic expression of hSNF5 inhibits cell growth and induces cellular senescence (Oruetxebarria et al., 2004). It was found that hSNF5 exerts these effects via the p16^INK4a^/Rb pathway, as the re-expression of hSNF5 in MRT cells activated *INK4b* and *INK4a*, but not *ARF* (Chai et al., 2005; Kia et al., 2008). hSNF5 activates *INK4a* in these cells by recruiting the SWI/SNF complex to its promoter. As a result of its recruitment, the PRC1 and PRC2 complexes are displaced from the promoter (Kia et al., 2008).

### Transcription factors involved in the regulation of INK4/ARF locus

**FIGURE 3:**
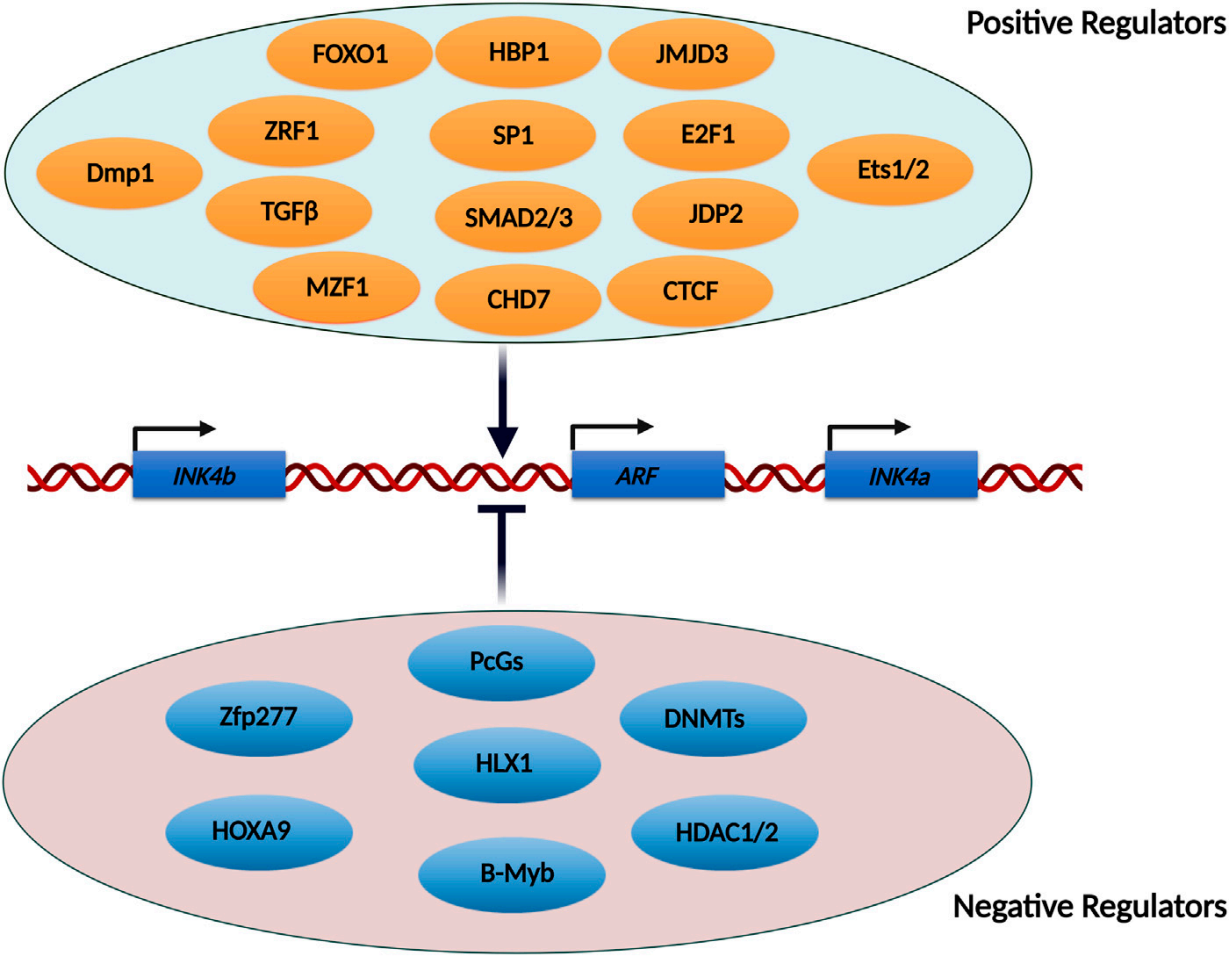
List of transcription factors and epigenetic modifiers known to regulate the INK4/ARF locus. The INK4/ARF locus is regulated by a number of transcription factors and epigenetic modifiers. Some of the factors stimulate transcription from this locus, whereas others repress it. Some of these factors act directly on the promoters, facilitating the binding of RNA polymerase, while others activate enhancers located upstream of the genes.

Many transcription factors have been implicated in the regulation of the INK4/ARF locus. The majority of these transcription factors have been shown to act directly on the promoters of these genes. A few of them have been found to operate via upstream enhancer elements. While several transcription factors are required for the activation of the *INK4/ARF* genes, some have been demonstrated to inhibit their transcription (Figure 3). Due to the limited scope of this review, we have described only a few transcription factors involved in the activation of this locus.

**FOXO1.** FOXO1 is a tumor suppressor protein that inhibits Myc-induced lymphomagenesis in mice by activating the *ARF* gene. FOXO1 directly regulates *ARF* expression by binding to a motif located in the intron between exon1β and exon1α (Bouchard et al., 2007).

**ZRF1.** Zuotin-related factor 1 (ZRF1), a ubiquitin recognition domain-containing transcription factor, promotes the expression of PRC1-repressed genes during differentiation by competing for H2AK119Ub with PRC1 (Richly et al., 2010). ZRF1 expression is enhanced in MEFs and is recruited to the *INK4/ARF* promoters following hRas overexpression. Ectopic expression of ZRF1 activates *INK4a* and *INK4b* in IMR90, but not ZRF1delUBD, showing that ZRF1 binding to H2AK119Ub is necessary for its recruitment during senescence. (Ribeiro et al., 2013).

**Dmp1.** The deletion of Dmp1, a well-characterized tumor suppressor, accelerates tumor growth in mice. It acts as a link between Ras/Raf overexpression and *INK4/ARF* gene activation. Dmp1 expression is promoted by Ras overexpression, and it enhances *ARF* transcription by directly binding to Dmp1/ETS motif present in its promoter (Sreeramaneni et al., 2005).

**JDP2.** Jun dimerization protein 2 (JDP2) is a transcription factor that binds to JDP2 response regions and prevents histone acetylation and methylation (Huang et al., 2011). JDP2 is required for normal cell differentiation and proliferation, as MEFs lacking JDP2 do not undergo replicative senescence. Its overexpression inhibits MEF proliferation by increasing the expression of *INK4a* and *ARF* (Nakade et al., 2009).

**CTCF.** In U2OS cells, CTCF binds to a DNA sequence near the *ANRIL* promoter, and its silencing results in down-regulation of all three *INK4/ARF* genes. CTCF binding is lost when its DNA motif is methylated, resulting in the downregulation of these genes (Rodriguez et al., 2010).

**CHD7.** Chromodomain helicase DNA binding protein 7 (CHD7) is an ATP-dependent chromatin remodeler that plays a critical role in Ras-mediated senescence. It is essential for the activation of *INK4a* following Ras overexpression (Su et al., 2018). Transcription factors like c-Jun and Ets1 promote Myeloid zinc finger 1 (MZF1) expression during Ras-induced senescence, which in turn recruits CHD7 to the promoter of *INK4a* for its upregulation (Wu et al., 2022).

**HBP1.** HMG box-containing protein 1 (HBP1) transcription factor is a downstream effector protein in the Ras signaling pathway. *INK4a* promoter contains a putative binding motif for this transcription factor between positions −426 and −433. Its binding to this motif triggers cellular senescence (Li et al., 2010). HBP1 promotes acetylation of the *INK4a* promoter by assisting in the recruitment of histone acetyltransferase p300 and CREB-binding protein (CBP) (Wang et al., 2012). Furthermore, ectopic expression of HBP1 induces premature cellular senescence in normal fibroblasts via *INK4a*, while its knockdown delays senescence and senescence-associated phenotypes (Wang et al., 2012).

**SP1.** *INK4a* promoter has numerous GC-rich regions that are required for its induction upon senescence onset (Wu et al., 2007). SP1, a transcription factor, with a strong affinity for GC-rich motifs binds to these regions to enhance *INK4a* expression. In human fibroblasts, ectopic expression of SP1 upregulates the *INK4a* (Wu et al., 2007). Furthermore, SP1, like HBP1 physically interacts with p300/CBP to promote *INK4a* expression. (Wang et al., 2008).

### Transcriptional regulation of INK4/ARF locus by distal regulatory elements

As stated earlier, the gene desert region upstream of the *CDKN2A/B* genes contains several SNPs that are strongly associated with the risk of CAD and type 2 diabetes in humans. In mice, deletion of this CAD (70 kb) interval resulted in a substantial decrease in the cardiac expression of *CDKN2A/B* genes, significantly increased mortality upon high cholesterol diet and ARF-dependent developmental abnormalities (Visel et al., 2010). Primary cells isolated from such mice showed increased proliferation compared to wild-type cells and exhibited no signs of senescence over subsequent passages (Visel et al., 2010). Furthermore, allele-specific expression analysis in heterozygous mice carrying a CAD interval deletion on one chromosome revealed that the *cdkn2b* gene was preferentially expressed from the allele with a wild-type CAD interval, but the expression of the allele bearing the CAD deletion was dramatically reduced in the heart and other organs, implying that CAD interval may regulate these genes through a distant-acting cis-regulatory mechanism (Visel et al., 2010). Further work indicated that the mice lacking the CAD interval developed primary vitreous hyperplasia at the E13.5 developmental stage. It is well established that TGFβ regulates *ARF* expression in developing eyes and MEFs (Freeman-Anderson et al., 2009). It leads to *ARF* induction in MEFs and HeLa cells (Zheng et al., 2010).

In pursuit of understanding how this interval regulates the expression of *INK4/ARF* genes and to biologically underpin the genetic variations in the interval seen in several diseases, the interval was tested for the presence of distal regulatory elements known as enhancers. Towards this, a landmark study established the presence of several enhancers in the gene desert region of this locus (Harismendy et al., 2011). A relationship between the CAD-associated genetic variations (rs10811656 and rs10757278) in one of the enhancer elements (ECAD9) where STAT1 binds upon IFNγ stimulation was established. STAT1 binding on the homozygous CAD risk allele was reduced in lymphoblastoid cells (LCL) therefore, the knockdown of STAT1 in LCLs that were homozygous for the non-risk CAD allele upregulated *CDKN2BAS* suggesting a repressive role of STAT1 on *CDKN2BAS* expression. However, HUVEC cells exhibited an activatory role of STAT1 on the expression of *CDKN2BAS* suggesting, the effects of CAD risk allele on *INK4/ARF* genes could be cell-type specific (Harismendy et al., 2011). Notably, the CAD risk interval contains a cis-acting enhancer that collaborates with TGFβ to promote *ARF* expression during development (Zheng et al., 2013), and mice lacking the CAD interval don’t show such induction of *ARF*, implying that TGFβ works on *ARF* via the enhancers in CAD interval. Furthermore, TGFβ induces three H3K27ac peaks at the 110 kb distance from the *CDKN2A* promoter in HeLa cells and the deletion of a 20 kb area spanning all three peaks significantly lowers *ARF* and *INK4b* expression (Liu et al., 2019). These findings imply that TGFβ affects the transcription of these genes by activating the enhancers upstream of the genes (Liu et al., 2019). Macrophages derived from mice with an atherosclerosis susceptibility locus express significantly lower levels of *INK4a* and *ARF* (p19 in mice), but not *INK4b* (Kuo et al., 2011). Furthermore, individuals with the risk allele rs10757278, which has been related to an increased risk of atherosclerosis, have lower expression of all three *INK4/ARF* genes and even ANRIL in peripheral blood T-cells (Liu et al., 2009). Another study discovered a cis-regulatory region adjacent to the *ARF* promoter that represses *INK4a* gene expression. This element loops with the promoter of *INK4a* to repress its transcription. Perturbation of the element stimulated the transcription of the *INK4a* gene. (Zhang Y et al., 2019). All of these studies show a connection between disease-associated SNPs in the gene desert interval and *INK4/ARF* gene expression. Taken together, the risk alleles for CAD and atherosclerosis are primarily associated with lower expression of the *INK4/ARF* genes and these effects are cell-type specific.

**FIGURE 4:**
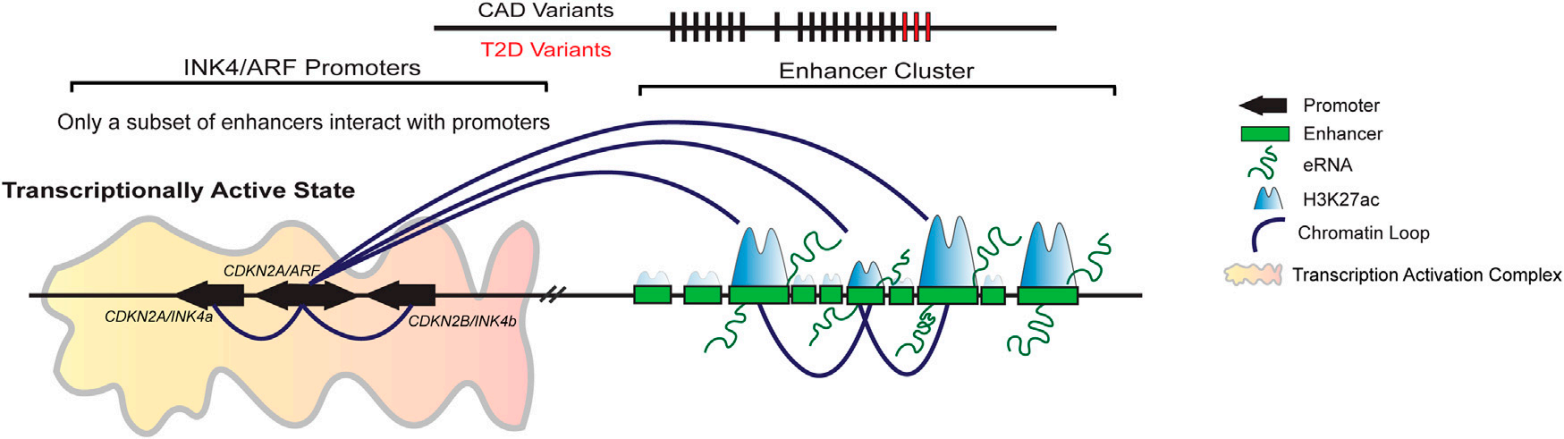
A subset of enhancers in the enhancer cluster upstream regulates INK4/ARF locus. Gene desert upstream of *INK4/ARF* genes contains 21 enhancers in HeLa cells. Only 15 of these enhancers are active, displaying both H3K27ac and H3K4me1 marks. Out of 15 active enhancers, only a subset of enhancers interacts with the promoters of *INK4/ARF* genes. The promoter interacting enhancers are critical for the regulation of these genes. The deletion of a single enhancer causes down regulation of gene transcription and EZH2 loading on the promoters. Furthermore, the deletion of interacting enhancers has an effect on the other enhancers in the cluster, indicating that the enhancers are interdependent.

Recently, we showed that the gene desert region upstream of the *INK4/ARF* genes contains 21 potential enhancer elements in the HeLa cells. Among these enhancers, 15 enhancers exhibited marks of active enhancers such as H3K27ac, PolII, and eRNA transcription. Out of these, only five active enhancers interacted with the *CDKN2A/B* gene promoters. However, disruption of any of these interacting enhancers but not non-interacting enhancer impacted the expression of *INK4a*, *ARF*, and *INK4b* at similar levels (Farooq et al., 2021). Interestingly, the interacting and non-interacting enhancers were indistinguishable at the levels of enhancer marks such as levels of H3K27ac, p300, and eRNA expression. This indicates that the bio-chemical marking of enhancers alone fails to predict enhancer activity (Farooq et al., 2021) (Figure 4). However, how SNPs in these enhancers regulate the locus in various diseases requires more efforts focused on functional studies to molecularly underpin the genetic variation and associated diseases in this locus. The resultant mechanistic understanding will pave the way for future therapeutic interventions.

### LncRNAs as transcriptional regulators of the INK4/ARF locus

**FIGURE 5:**
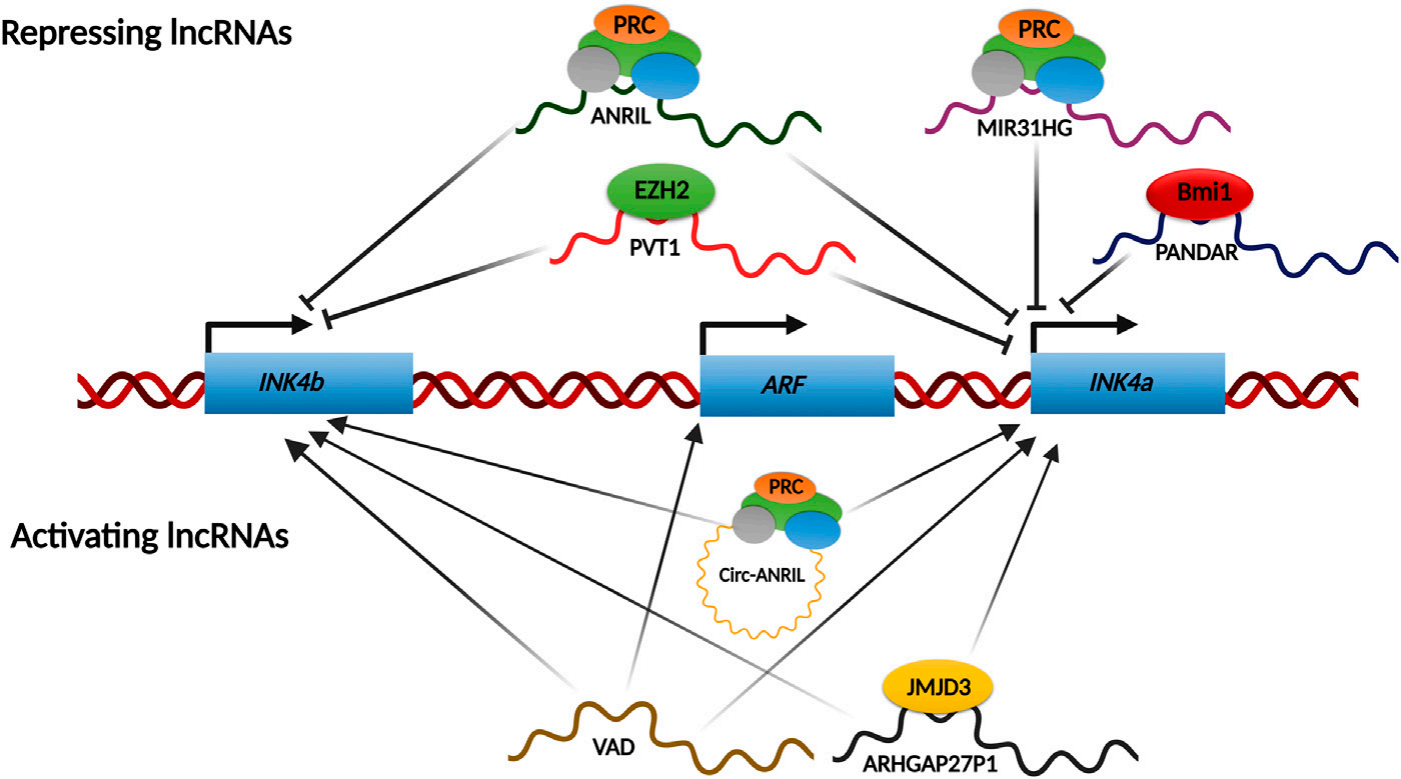
lncRNAs network regulating INK4/ARF locus. Various lncRNAs regulate the INK4a/ARF locus under certain conditions. Most of the known regulatory lncRNAs shut down the transcription from this locus by recruiting PcGs onto the promoters. However, some lncRNAs have been shown to activate this locus transcriptionally by either recruiting chromatin modifiers such as JMJD3 onto the promoters or by removing the repressive complexes like PcGs from the promoters.

Long noncoding RNAs (lncRNAs) are a subclass of RNAs that are longer than 200 nucleotides and do not code for any protein product. They play critical roles in gene regulation, chromatin organization, translational regulation, etc. Several lncRNAs have been reported to influence the INK4/ARF locus expression (Puvvula 2019). Most of them are repressive and act by recruiting the PcG complexes onto the promoters of these genes. Recently, certain lncRNAs have been described to activate this locus (Figure 5).

### LncRNAs-mediated repression of INK4/ARF locus

INK4/ARF locus contains a lncRNA, ANRIL, which is transcribed antisense to the genes. ANRIL is ∼3.8 kb and has over 20 different splice variants. These splice variants of ANRIL play a differential protective role depending on the presence or absence of CAD risk interval (Lo Sardo et al., 2018). ANRIL is required for *INK4/ARF* silencing in growing cells, as its expression in these cells is inversely correlated to gene expression (Yap et al., 2010; Kotake et al., 2011). This repression is a result of PRC2 loading on the *INK4a* promoter by nascently transcribing ANRIL RNA (Yap et al., 2010). Another study demonstrated similar recruitment of PRC2 to the *INK4b* promoter (Kotake et al., 2011). Therefore, ANRIL expression decreases as senescence progresses for the activation of this locus. Through RNA binding experiments, ANRIL was shown to interact with the CBX7 component of PRC1 to enhance *INK4/ARF* gene silencing (Yap et al., 2010). Subsequently, ANRIL binding to SUZ12, a component of the PRC2 complex, was shown to enhance the silencing of *INK4b*, but not *INK4a* (Kotake et al., 2011). In contrast to these observations, ANRIL expression is positively linked with *INK4a/ARF* expression, in cervical cancers. In a recent study, we report that the PRC2 complex can bind to the *INK4/ARF* promoters independent of ANRIL levels in cervical cancer cell lines (Farooq et al., 2021). Another lncRNA, MIR31HG, which is transcribed from the short arm of chr9 itself, has been shown to recruit PRC complex on the *INK4a* promoter (Montes et al., 2015). Interestingly, during OIS (Oncogene induced senescence), MIR31HG localizes solely to the cytoplasm. This leads to the loss of the PRC complex from the *INK4a* promoter, resulting in transcriptional activation of *INK4a* (Montes et al., 2015). PANDAR (promoter of CDKN1A antisense DNA damage-activated RNA) is elevated in breast cancer tissues and cell lines. PANDAR interacts with Bmi1, a PRC1 subunit, inhibiting *INK4a* transcription by loading Bmi1 to its promoter. PANDAR silencing reduces cell proliferation and colony formation in MCF7 cells and causes G1/S arrest in a p16^INK4a^-dependent manner (Sang et al., 2016). PVT1 is critical for gastric cancer progression. It accomplishes this in part by forming a complex with EZH2 and directing it to the promoters of *INK4b* and *INK4a*, suppressing their expression in gastric cancer (Kong et al., 2015). ANROC, a recently discovered lncRNA, is found downstream of the *INK4a* gene, and its silencing results in overexpression of all three genes, indicating that ANROC is a repressive RNA (Kotake and Tsuruda 2020).

### LncRNAs-mediated activation of INK4/ARF locus

LncRNA ARHGAP27P1 is downregulated in gastric cancer cells, and when overexpressed, it inhibits gastric cancer cell proliferation, migration, and other functions in a p16^INK4a^ and p15^INK4b^- dependent manner. This lncRNA regulates *INK4/ARF* expression by interacting with and directing the histone demethylase JMJD3 to the promoters for removal of the repressing H3K27me3 mark (Zhang G et al., 2019). AUF1 is an RNA-binding lncRNA that has been found to enhance the degradation of various RNAs. P14AS was identified using RNA capture sequencing as a novel RNA with its promoter located on the antisense strand of the fragment near *CDKN2A* exon1β. P14AS binds to AUF1, preventing ANRIL/INK4a RNA from interacting with AUF1. This competitive interaction between P14AS and AUF1 promotes *ARF*, *INK4b*, and *INK4a* gene expression (Ma et al., 2020). During OIS, VAD (Vlinc RNA Antisense to DDAH1) is highly upregulated and required to maintain senescence characteristics. VAD functions in trans on the INK4/ARF locus, and its depletion causes downregulation of *ARF*, *INK4b*, and *INK4a*. VAD promotes the expression of these genes by removing H2A.Z from their promoters. H2A.Z deposition represses these genes by promoting the recruitment of the PRC complex to the promoters (Lazorthes et al., 2015). Several circular ANRIL isoforms have been identified that activate the *INK4/ARF* genes rather than inhibiting them. They switch from repressors to activators of these genes during RAF1-mediated senescence. These circular isoforms engage with Polycomb subunits and displace EZH2 from the *INK4b* and *INK4a* promoters, stimulating transcription of these genes. As a result of the PRC2 dislocation, H3K27me3 levels at these promoters drop (Muniz et al., 2021). Similarly, TUBA4b is downregulated in CRC tissues and cells, and its overexpression inhibits CRC cell proliferation by upregulating *INK4a* and *INK4b* (Zhou, Sun, and Zhou 2020). The precise mechanisms by which TUBA4b long noncoding RNA activates these genes are unknown.

## Discussion

Since the products of the *INK4/ARF* genes are implicated in a wide range of cancers and age-related diseases, they hold immense promise for treating or mitigating the consequences of these diseases. Regulation of the INK4/ARF locus is multi-layered, with a plethora of factors involved. As a result, the greatest challenge in harnessing this locus for therapeutic purposes is identifying the critical regulatory elements that can be targeted in a particular disease. Targeting transcription factors or epigenetic modifiers involved in its regulation has very broad effects, affecting not just this locus but others as well. We recently uncovered a few enhancers in the upstream enhancer cluster that regulate these genes. These DNA regulatory elements can be altered to provide a more precise and targeted effect. Since this enhancer cluster contains multiple enhancers, these enhancers may act in a tissue type-specific manner. Thus, determining which enhancers regulate these genes under various physiological conditions is critical for the therapeutic use of enhancers or enhancer products like eRNAs. Additionally, SNPs in upstream enhancer regions have been associated with several diseases. These SNPs can facilitate the identification of regulatory enhancers in various cell types. Thus, a functional genomics approach is required to decipher how these SNPs result in changes in gene regulation.
